# Paths to a malaria vaccine illuminated by parasite genomics

**DOI:** 10.1016/j.tig.2014.12.005

**Published:** 2015-02

**Authors:** David J. Conway

**Affiliations:** Pathogen Molecular Biology Department, Faculty of Infectious and Tropical Diseases, London School of Hygiene and Tropical Medicine, Keppel Street, London WC1E 7HT, UK

## Abstract

More human death and disease is caused by malaria parasites than by all other eukaryotic pathogens combined. As early as the sequencing of the first human genome, malaria parasite genomics was prioritized to fuel the discovery of vaccine candidate antigens. This stimulated increased research on malaria, generating new understanding of the cellular and molecular mechanisms of infection and immunity. This review of recent developments illustrates how new approaches in parasite genomics, and increasingly large amounts of data from population studies, are helping to identify antigens that are promising lead targets. Although these results have been encouraging, effective discovery and characterization need to be coupled with more innovation and funding to translate findings into newly designed vaccine products for clinical trials.

## Highlights

• Discovery of vaccine candidate antigens by parasite genome sequence analyses.
• Genetic crosses, linkage group selection, and functional studies on parasites.
• Characterizing developmental and epigenetic variation alongside allelic polymorphism.
• Selection by naturally acquired immune responses helps to focus vaccine design.

## The need for a malaria vaccine

The drive to sequence malaria parasite genomes began in the 1990s with a bold vision for ‘vaccinomics’ which would showcase the benefits of genomics to society [1]. This was well conceived, and remains relevant because malaria causes at least half a million deaths and 200 million clinical cases each year, with several species of *Plasmodium* parasites infecting humans via transmission by *Anopheles* mosquitoes [2]. The disease occurs in most tropical areas of the world, with the greatest burden being on children and pregnant women in Africa where *Plasmodium falciparum* is most prevalent. Existing interventions need to be more widely applied, including insecticide-treated bed nets to reduce mosquito biting, antimalarial drug prophylaxis targeted to vulnerable groups, and access to prompt diagnosis and treatment. An effective vaccine could be a cost-effective additional means of controlling malaria [3] and might enable its elimination from some areas.

A key issue for malaria vaccine design is selecting the most appropriate parasite life-cycle stage to be targeted (Box 1) [4,5]. A vaccine to prevent blood-stage infection from occurring could target the parasite at the initial pre-erythrocytic stage, but would need to be fully effective because even a small number of parasites emerging from the liver can initiate a severe blood-stage infection. By contrast, a vaccine targeting the asexual blood-stage directly could be effective in suppressing the replicating parasites and thereby preventing most disease, even if it did not achieve absolute sterile immunity. As a potential complement to either of these, vaccination against parasite sexual stages would not prevent disease directly but might reduce transmission to mosquitoes, thereby possibly having a beneficial effect at the population level. Molecular characterization of these developmentally differentiated stages was an early goal of genomic and transcriptomic studies.

**Figure 1:**
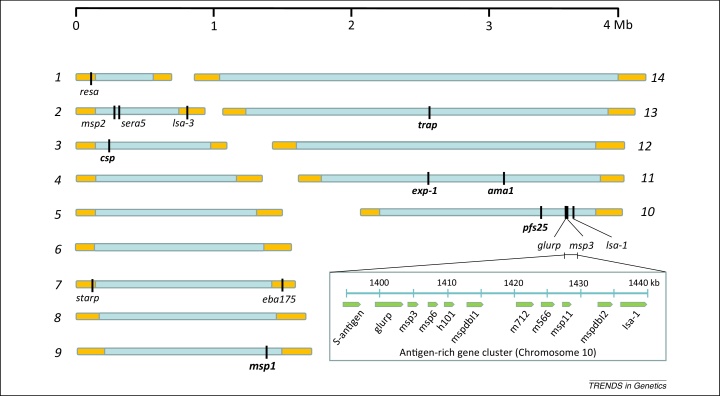
Haploid *Plasmodium falciparum* genome containing 14 chromosomes. The whole genome of 23 Mb contains ∼5500 protein-coding genes. Subtelomeric regions shaded orange are highly divergent in sequence and gene content among species; the core genome shaded blue has a high level of synteny among *Plasmodium* species, although some genes are species-specific. Loci encoding the 15 antigens incorporated in reported clinical vaccine trials are shown. Only six of these have clear orthologs in all *Plasmodium* species (shown in bold font). The full names of each of these antigens are given in Box 2. A region of chromosome 10 is enlarged on the bottom right of the figure to show a cluster of antigen-encoding genes including several that are highly polymorphic (most are members of the *msp3*-like gene family flanked by unrelated antigen genes at both ends of the cluster).

The first genome sequence of a malaria parasite was published in 2002 for a cultured strain of *P. falciparum* containing approximately 5500 genes encoded on 14 chromosomes with a complete haploid genome size of ∼23 Mb [6] (Figure 1). Genome sequences of diverse malaria parasite species, including *P. vivax*, the second most important cause of human malaria [7], the zoonotic *P. knowlesi* [8], other primate malaria parasites [9,10], and parasites of rodents which can be studied in laboratory mice [11], show similar core genome organization. Each of the chromosomes shows a largely conserved syntenic gene order [12], except in the subtelomeric regions which contain large gene families that have diverged substantially between species [10,12,13]. Within each species, meiotic recombination occurs in a brief diploid stage within the mosquito midgut after fertilization between haploid male and female gametes in the blood meal. Recombination occurs at an average of 1 cM per 10–30 kb in experimental crosses of *P. falciparum* [14], although effective recombination rates in natural populations can be lower due to self-fertilization [15]. The diversity of parasites within infections directly influences the rate of outcrossing, which is greatest in highly-endemic populations where superinfection by unrelated genotypes commonly occurs [16]. Subtelomeric chromosomal regions can additionally undergo recombination during mitotic replication in the asexual blood stage of infection, with crossing-over occurring between non-homologous chromosomes in the same genome [17]. For vaccine design, the genetics and development of the parasite need to considered in the context of host immune responses that might suppress or eliminate an infection.

## Feasibility of vaccination

A degree of immunity to malaria is generally acquired by experience of the infection, as shown by experimental studies in the first half of the 20th century on patients with neurosyphilis. Fevers induced by administering malaria parasite infections were beneficial to such patients in the age before antibiotics, because elevated temperatures killed spirochetes more effectively than other available treatments, but many patients became immune to repeated malaria infections [18]. Studies on naturally acquired immunity in endemic populations then showed that malaria parasite levels in children or non-immune adults were reduced by passive transfer of serum Immunoglobulin G (IgG) from relatively immune adults, which has encouraged subsequent focus on the role of antibodies in acquired immunity to blood-stage parasites [19]. More detailed studies over the past 40 years have shown that immunization of human volunteers with attenuated parasites confers strong protection against experimental challenge infections [20,21].

Parasites may be attenuated at the infective sporozoite stage which invades hepatocytes [20], or at the subsequent blood stage in erythrocytes [21]. Intravenous vaccination using sporozoites isolated from irradiated mosquitoes requires a demanding regimen of five doses of >100 000 parasites over a 3 month period to elicit responses that prevent parasites developing beyond the pre-erythrocytic liver stage [22]. It is difficult to standardize the irradiation dose that each individual parasite receives, but alternative possibilities are chemical attenuation (e.g., alkylation of parasite DNA) [23], or genetic attenuation to remove the need for treatment of each batch. Multiple gene knockouts have been identified that, in combination, cause parasites to arrest development within the liver and elicit pre-erythrocytic stage immunity [24,25]. Engineering parasites to attenuate at the blood stage would require modification of metabolism to allow growth in selective laboratory media but not in normal human blood, as illustrated by parasites that have lost the apicoplast organelle under selection in culture [26,27].

Although there is a precedent for use of live-parasite vaccines to prevent protozoan infections of domestic animals [28], subunit vaccines containing specific antigens are generally considered more feasible for broad delivery to human populations [4,5]. These may be constructed as recombinant proteins (that are either soluble, or form virus-like particles, or chemically conjugated nanoparticles), long synthetic peptides, recombinant plasmid DNA, or recombinant viral vectors, each potentially offering a product profile that could be incorporated into an Expanded Program for Immunization (EPI) schedule [29]. The most advanced candidate developed to date is the RTS,S vaccine, which includes a large portion of the primary sequence of *P. falciparum* CSP (circumsporozoite protein – see Box 2 for abbreviations and names of all malaria antigens subsequently referred to) sequence co-expressed with the hepatitis B surface antigen as a virus-like particle, delivered with a potent proprietary adjuvant (ASO1). This shows safety and short-term protective efficacy of 20–50% against malaria infection and disease in most clinical trial populations [30]. The experience of RTS,S and other recombinant protein vaccine development indicates the importance of adjuvant choice to elicit high-titer antibodies. Viral-vectored vaccine delivery may be optimized through prime–boost schedules expressing malaria antigens by one recombinant virus (such as an adenovirus) in the first immunization and by an unrelated viral vector (such as a poxvirus) in boosting immunizations [31]. Some schedules incorporating particular candidate antigens show potent immunogenicity, although only a small minority of individuals have been protected from challenge infection and the determinants of efficacy need to be resolved [32]. However, for any subunit vaccine, inclusion of the most-effective target antigens is vital, and it is for their identification that parasite genomics is most important.

## Discovery of vaccine candidate antigens

**Figure 2:**
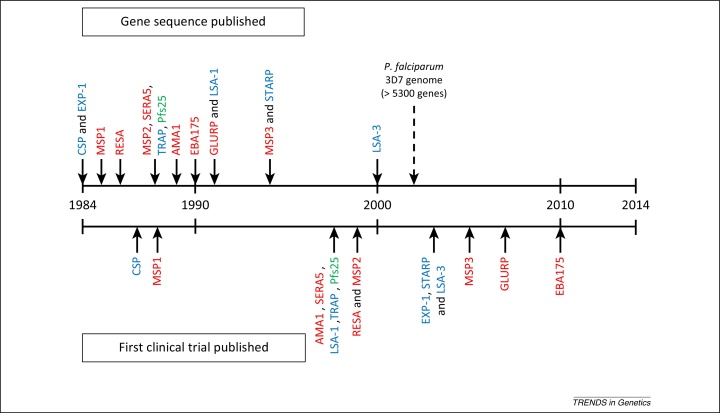
30-Year timeline of *P. falciparum* vaccine antigen sequence description and first clinical trial publication for each antigen. Dates of first description of gene sequences are shown by arrows above the timeline for each of the 15 *P. falciparum* antigens with sequences incorporated into vaccines reported in published clinical trials (arrows below the timeline indicate the first trial in each case). The color shading indicates the parasite life-cycle stage at which each of the antigens is mainly expressed: pre-erythrocytic (blue), asexual blood stage (red), sexual and zygote transmission stage (green). The nature of the vaccine constructs varied extensively, based on synthetic peptides, recombinant proteins, virus-like particles, or viral vectored systems. In some cases only a short sequence representing part of an antigen was included in the vaccine, combined with sequences from other antigens, whereas in other cases most of a primary sequence of a single antigen was incorporated. For several of the antigens, other vaccine constructs have also been designed and tested at later dates that are not shown on the scheme. The date of publication of the first parasite genome sequence in 2002 is marked by a broken arrow above the dateline.

Vaccine candidates based on 15 different antigens of *P. falciparum* have proceeded to clinical trials that have been published so far (Figure 1 and Box 2). These antigens are diverse proteins, expressed at various stages of the life cycle, and were first described during an early period of parasite molecular characterization; the sequences of these genes were published between 1984 and 2000 when most other genes of the parasite were still unknown (Figure 2). It is remarkable that there have been no reported vaccine trials incorporating any of the thousands of proteins encoded by genes first described in the reference parasite genome sequence published in 2002 [6]. It is very improbable that all promising candidate antigens were discovered ahead of the genome sequence, and some potential reasons for the lack of vaccine trials based on genomic findings are discussed below.

For the 15 vaccine candidate antigens that have undergone trials to date, there was a mean overall ‘lag period’ of 10 years from the primary publication of each antigen gene sequence until the first publication of a clinical vaccine trial incorporating a sequence of the respective antigen (Figure 2). There was a wide range in this time-window (from only 3 years for CSP, MSP1, and LSA-1, to 20 years for EBA-175), and for 11 out of the 15 antigens the period was less than 12 years. This shows that it is possible, as illustrated by most of these examples, to proceed from initial antigen gene discovery to a reported vaccine trial in less time than has already elapsed since the first malaria parasite genome publication in 2002. The absence of any published vaccine trial incorporating a new candidate antigen discovered in the genome sequence is therefore not a trivial observation. Another possible explanation is that most of the limited capacity and funding for malaria vaccine development may be committed to supporting previously identified candidates. Focus on a limited pre-existing portfolio might have unintentionally blocked new candidate development, even without proprietary or other conflicting interests which could exacerbate such an effect. A further possibility is that that the increased scale of discovery promoted by genome sequence analysis has itself inhibited the selection of candidates because there are now so many proteins to investigate that there may be increased uncertainty about the type and amount of data required for prioritization. Clearly, vaccine candidates should not continue to be prioritized for product development solely on the basis of the order in which they were discovered, and there is therefore a need for an evidence-based process to mark the candidacy of an antigen. Given the complexity of the parasite life cycle, and the diverse immune mechanisms that may be relevant, standardization will not be simple, but the following section outlines relevant evidence for inclusion.

## Evidence to prioritize candidate antigens

### Profiling naturally acquired immune responses

Studies to identify specific human immune responses associated with protection from malaria are important, particularly when responses to multiple antigens are analyzed in parallel. These involve laboratory assays of antigen-specificity of naturally acquired serum antibodies, or peripheral blood lymphocytes in culture, and correlations with incidence of clinical malaria of the sampled individuals in cohort studies of endemic populations [19,33–35]. The incidence of clinical malaria is more relevant to measure than the incidence of any detected infection by the parasite because naturally acquired immunity mainly acts to reduce the density of parasites in the blood rather than entirely preventing infection [36,37]. A separate approach has been to analyze immune responses in individuals protected by experimental immunization with attenuated parasites, screening reactivity against various parasite proteins in an attempt to implicate particular specificities [38]. The difficulty in producing high-quality recombinant antigen reagents has limited the effectiveness of protein microarrays to scale up to the parasite proteome, and thus prioritization of antigens for analysis is ideally determined by predictions from genetic and functional studies.

### Genetic crosses and linkage group selection

Experimental genetic crosses of parasites have been vital in identifying genes that encode targets of immunity [39], determinants of virulence [40], erythrocyte invasion [41], and drug resistance [42]. They have been most extensively applied in studies of rodent malaria parasites, particularly *P. yoelii* [40] and *P. chabaudi* [42]. Although some studies require cloning of progeny, it is also possible to analyze uncloned progeny to identify genomic regions influencing growth or survival under experimental conditions. This linkage group selection approach has identified loci encoding targets of strain-specific immunity in rodent malaria, confirming the importance of the merozoite surface protein MSP1 [39]. This approach may be widely applicable for genetic mapping of immune targets in other apicomplexan parasites, including *Eimeria* which causes coccidiosis in chickens [43].

It is notable that some important targets of immunity are encoded by genes occurring only in *P. falciparum* or *P. vivax* but not in rodent malaria parasites, including several of the vaccine candidates already developed (Figure 1). However, the linkage group selection approach has not yet been applied to identify targets of acquired immunity in human parasites directly. Very few genetic cross experiments have been published for *P. falciparum*, and none for *P. vivax*, because the procedure requires inoculating progeny parasites from mosquitoes to splenectomized chimpanzees to allow parasites to develop to blood stages. In one study, a key role of the *P. falciparum* merozoite ligand Rh5 (which does not have an ortholog in rodent parasites) in erythrocyte invasion was identified by mapping this locus for ability to invade *Aotus* monkey erythrocytes [41], and subsequent studies to characterize this potential target of immunity are noted below. Because experimental infections of chimpanzees may no longer be conducted, owing to ethical considerations, it would be relevant to consider studies of uncloned parasite progeny in human volunteers, under safety guidelines that have already been defined for experimental infections [44]. Genetic cross experiments on human malaria parasites may also utilize an immunodeficient mouse with adoptively transplanted human hepatocytes, allowing development of parasites from mosquitoes through the liver stage, after which they may be harvested into transfused human erythrocytes [45].

### Functional studies

Parasite genetic cross experiments map loci down to a few cM, typically in the order of approximately 50 kB which may contain upwards of 10 genes, after which targeted gene knockout and allelic replacement may identify causal genes. Large libraries of gene knockouts are now available for rodent malaria parasites that are most amenable to genetic manipulation [46], while targeted modification of *P. falciparum* has also undergone scale-up [47] and methodological innovation [48,49]. In conjunction with these approaches, protein–protein interaction studies have revealed functions of particular gene products, as illustrated by defining the role of Rh5 in merozoite invasion, after genetic mapping [41].

Experimental knockout of the *Rh5* gene in *P. falciparum* was not achieved despite repeated attempts, suggesting that it may be essential to parasite growth at the cultured asexual blood stage [50]. Prey–bait screening of protein interactions then revealed that Basigin is the erythrocyte surface receptor for Rh5 [51], a crucial finding that led to studies on antibody inhibition of the binding [52,53] and inhibition of erythrocyte invasion [54], as well as structural analysis of the molecular interactions [55]. The relative abundance of *Rh5* transcript at the merozoite-containing schizont stage appears to vary among parasite isolates from clinical cases [56], but anti-Rh5 antibodies significantly inhibit merozoite invasion by all isolates tested so far [52]. Naturally occurring amino acid polymorphisms in Rh5 do not affect human Basigin receptor binding or antibody inhibition [52], but several mutations that alter Rh5 binding to *Aotus* monkey erythrocytes have occurred in a parasite line adapted to these hosts [57]. Another merozoite protein, termed the Rh5-interacting protein (Ripr), has been subsequently identified and is being analyzed as a potential target of immunity [58]. The prey–bait system that identified the parasite–host Rh5–basigin interaction allows broad screening, and has already identified merozoite protein MTRAP as a ligand binding to semaphorin-7A on erythrocytes, although antibodies were unable to block this particular interaction [59]. Apart from Rh5–Ripr, other key interactions between parasite proteins include a functional complex between the candidate antigen AMA1 and rhoptry neck protein 2 (RON2) at the apical tip of merozoites during erythrocyte invasion, and immunization of animals with both components has elicited more inhibitory antibodies to AMA1 than was achieved by immunization with AMA1 alone [60].

### Characterizing developmental and epigenetic variation

Expression of *P. falciparum* genes at particular life-cycle stages has been described by transcriptome profiling, with particular detail for the cultured 48 h cycle of the asexual blood stages [61,62]. Apart from stage-specificity, which is largely orchestrated by a family of AP2 transcription factors [63], many genes show clonally variant expression among individual parasites at a particular stage [64] (Box 3). A survey of asexual blood stages of several laboratory clones and subclones of *P. falciparum* estimated that approximately 10% of all genes exhibit clonally variant transcription [65]. Detection of expressed protein in individual parasites also reveals clonal variation that can be hard to resolve by analysis of transcript levels in bulk-cultured populations [66].

Malaria parasite genes in subtelomeric chromosomal regions show extreme patterns of sequence diversity and clonal expression variation, particularly the *var* gene family with approximately 60 copies in each haploid *P. falciparum* genome encoding variants of the parasite-infected erythrocyte membrane protein 1 (PfEMP1) [6]. The antigenic repertoire of PfEMP1 is too large to allow development of a multivalent vaccine to cover the existing diversity, but two lines of investigation support the concept of a vaccine based on a small subset of antigenic types. First, parasites which adhere to the receptor chondroitin sulfate A (CSA) on placental capillary endothelia in pregnant women express a particular variant of PfEMP1, termed VAR2CSA, that is encoded by only one or a few similar *var* gene types in each parasite genome [67,68], although there is considerable antigenic diversity due to allelic polymorphism within populations. A vaccine which either covered VAR2CSA polymorphism within a multi-allelic formulation or which focused responses on conserved epitopes might protect against pregnancy-associated malaria, and this prospect has led to structural studies to design proteins based on particular domains eliciting antibodies that inhibit receptor binding [69,70]. A second aspect of *var* gene specialization is that the expression of particular genes is associated with severe clinical forms of malaria in children [71–73], probably due to variant pfEMP1 products causing infected erythrocyte cytoadhesion to particular capillary endothelial surfaces [74,75] or enhanced rosetting of uninfected erythrocytes around infected erythrocytes [76]. Antigenic cross-reactivity among candidate PfEMP1 sequences is being studied towards identifying a subset of candidate products for a multivalent vaccine against severe malaria [77].

**Figure 3:**
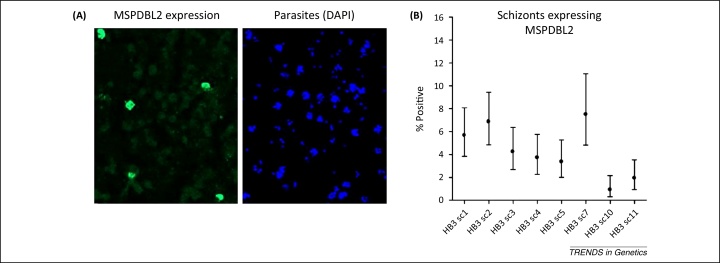
Example of variation in expression of a malaria parasite antigen. The *P. falciparum* MSPDBL2 protein is encoded by an *msp3*-like gene expressed on merozoites contained in mature blood-stage schizonts. The expression is variable among individual parasites because most schizonts are negative whereas a minority are positive. The figure shows example data from one parasite clone (HB3) (reproduced with modification from [66]). **(A)** A single microscopy field showing fixed parasite-infected erythrocytes. The left panel shows immunofluorescence of schizonts that react with an anti-MSPDBL2 antibody; the right panel shows DAPI (4′,6-diamidino-2-phenylindole)-stained parasite DNA in the cells (schizonts having multiple blue clustered nuclei). **(B)** Proportions of mature schizonts positive for MSPDBL2 vary among different subclones of the HB3 parasite. The eight subclones shown were cultured separately for several weeks, and then analyzed by microscopy counting of hundreds of mature schizonts from each to determine the percentage positive (with 95% confidence intervals).

Other large subtelomeric gene families in *P. falciparum* termed *rifin* and *stevor* encode proteins that are variably expressed on infected erythrocytes and merozoites, although whether antibodies to these are protective is not as well established as for PfEMP1 [78,79]. Merozoite proteins encoded by smaller gene families are also variably expressed, including several encoded by *msp3*-like genes clustered in a non-subtelomeric position of the genome [66] (Figure 1). For one of these proteins, MSPDBL2, only a minority of mature merozoite-containing schizonts within any parasite line are positive, and Figure 3 illustrates the variability among cultured subclones of a single parasite clone. Among members of the EBA and Rh families of merozoite proteins that bind alternative erythrocyte receptors for invasion, some reciprocal patterns of expression and function have been shown. In particular parasite clones, experimental knockout of the gene encoding the ligand EBA175 (which binds to glycophorin A on the erythrocyte surface) leads to increased expression of the alternative ligand Rh4 (which binds to complement receptor 1 on the erythrocyte surface), supporting a concept of vaccine design to incorporate both of the alternative ligands [80]. These candidates might be combined along with other alternatives, such as EBL-1 that is expressed in some parasites to bind to glycophorin B, or EBA140 that binds to glycophorin C [81], or alongside more broadly required merozoite proteins such as Rh5 [54] or its adjunct binding partner Ripr [80,82]. Several of these parasite ligands also contain allelic sequence polymorphisms that have potential adaptive significance (Box 3).

### Detecting signatures of natural selection on antigens

**Figure 4:**
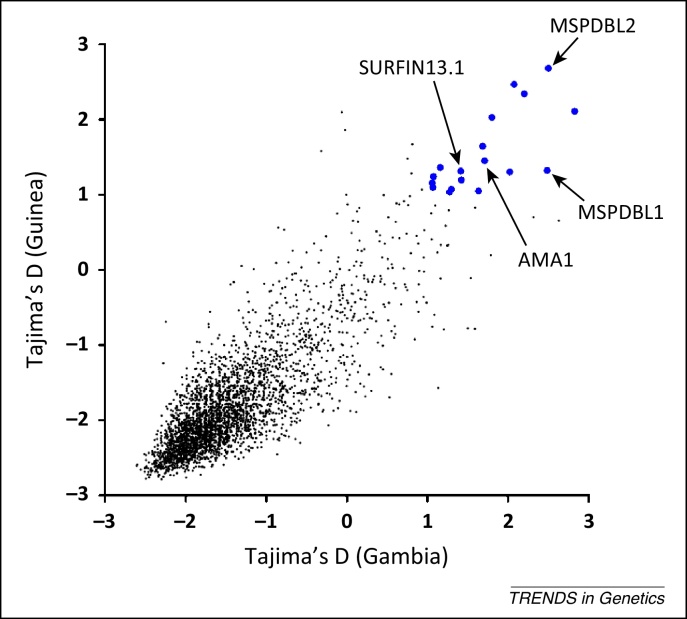
Genome-wide scanning for malaria parasite genes potentially under balancing selection. *P. falciparum* population genomic data from clinical isolates sampled from two different West African populations (Guinea and The Gambia) are plotted and compared. Each point represents a gene with at least three SNPs in each population (3316 genes in total included) on a scatterplot of Tajima's D values for the polymorphic site frequency spectra in Guinea (*n* = 100 isolates) and Gambia (*n* = 52 isolates) (reproduced with modification from [84]). This shows negatively skewed frequency distributions for most genes in both populations (with genome-wide average values of less than −1.0), indicating more rare alleles than expected under a neutral equilibrium, and probably reflects demographic growth of *P. falciparum* populations historically. Against this background, a minority of genes have positive Tajima's D values, indicating those at which allele frequencies are more balanced than expected under neutrality. Enlarged symbols are shown for the genes with values above 1.0 in both populations that are most likely to be under balancing selection. These include several known antigens that are labeled for illustration, together with other genes that have not been previously studied.

Pathogen antigens are likely to be subject to frequency-dependent selection from acquired immune responses, and particular patterns of allelic polymorphism may reveal departures from neutral expectations as a consequence. Several antigens already considered to be malaria vaccine candidates first showed such evidence [83], encouraging prospects that the identification of genes with similar patterns may reveal previously unidentified targets of immunity. Genome-wide scans of allele-frequency distributions in endemic West African populations have implicated particular genes as being under balancing selection, with alleles having more-intermediate frequencies than expected in the absence of selection, and in contrast with most of the genome [66,84] (Figure 4). This was particularly apparent for many genes with peak expression at the invasive merozoite stage, including members of the *eba*, *clag/RhopH1*, *surfin*, and *msp3-like* gene families, supporting the idea that this extracellular stage is particularly susceptible to acquired immunity [66,84]. Interestingly, a few of the antigen genes that are apparently under balancing selection may also have been under recent directional selection in local populations [85–87], suggesting that new alleles are occasionally introduced into the repertoire by mutation or immigration, and rapidly increase in frequency before being checked by allele-specific acquired immunity.

**Figure 5:**
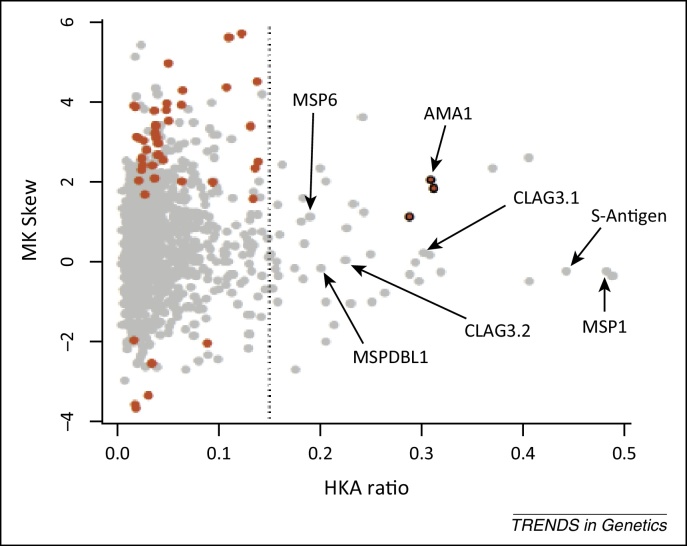
Genome-wide scan of gene polymorphism in *P. falciparum* versus divergence with the chimpanzee parasite *P. reichenowi*. Each point represents comparative data for two different indices (HKA ratio and MK skew) for a gene, with sequence data from five *P. falciparum* laboratory lines compared with a single *P. reichenowi* genome (reproduced with modification from reference [10]). Several known antigen genes are labeled for illustration. The HKA ratio for each gene (on the *x* axis) is the average pairwise nucleotide polymorphism in *P. falciparum* (π) divided by the average interspecies nucleotide divergence (*K*), with a broken line shown at an arbitrary ratio of 0.15 to visually indicate genes with highest relative levels of polymorphism on the right of the plot. The MK skew for each gene (on the *y* axis) is the log_2_-transformed odds ratio from the 2 × 2 table of numbers of nonsynonymous and synonymous polymorphisms within *P. falciparum* over the numbers of nonsynonymous and synonymous interspecies fixed differences. An MK skew value of zero represents an odds ratio of 1.0 reflecting where the intraspecific and interspecific ratios do not differ. Positive MK skew values indicate ratios for genes reflecting an excess of nonsynonymous polymorphism or deficit of nonsynonymous fixed differences compared with neutral expectations, marking in red those that are significant with Fisher's exact tests. Three of these genes that also have high HKA ratios are shown in bold outline (including AMA1). Some genes are not plotted here as they have infinite MK skew values (although significant in a number of cases by Fisher's exact test), mostly due to having no synonymous polymorphisms. Abbreviations: HKA, Hudson–Kretman–Aguade test; MK, McDonald–Kreitman test.

Genome sequences for the small number of *P. vivax* strains published to date indicate that this species is more polymorphic than *P. falciparum*, with nucleotide diversity being approximately twice as high, suggesting that this parasite has been widespread in humans for longer [88]. Analyses of *P. vivax* candidate antigen gene sequences in population surveys implicate *ama1*, *trap*, and *dbp* as being under balancing selection [89], consistent with results for their *P. falciparum* homologs [83]. To allow a provisional genome-wide scan for evidence of selection in parasite species for which there are only a few whole-genome sequences, information on polymorphism may be analyzed alongside comparative data on divergence between species. Contingency tests on polymorphism-versus-divergence ratios include the McDonald–Kreitman (MK) test for skew in the ratios of nonsynonymous and synonymous single-nucleotide polymorphisms (SNPs) and interspecies fixed differences, and the Hudson–Kretman–Aguade (HKA) test of the ratio of pairwise nucleotide polymorphism versus interspecies divergence, which may be compared across genes. This is illustrated for data on *P. falciparum* polymorphisms among a small number of laboratory lines compared with fixed differences between *P. falciparum* and the chimpanzee parasite *P. reichenowi* [10] (Figure 5). Genes with outlying values for MK and HKA ratios were largely independent, and known antigen genes more commonly had only high HKA ratios.

Genome-wide scans for loci under selection have not yet incorporated detailed analyses of repeat sequences, subtelomeric chromosome regions, or other loci with highly divergent alleles. Such sequences are not reliably characterized by short-read mapping to reference genomes and are therefore omitted from most analyses. However, advances in longer-read sequencing and methods of *de novo* assembly may soon capture the substantial polymorphism that exists in these regions [90], highlighting the need for suitable population genetic analytical methods for such loci, where processes of mutation and recombination do not fit simple models.

The predictive power of analyses to identify patterns of selection on antigen genes should be evaluated by epidemiological and experimental validation of the putative targets. In epidemiological studies, large cohorts of individuals need to be followed in order for levels of naturally-acquired antibodies against an array of different recombinant proteins to be tested for correlations with reduced prospective risk of clinical malaria [33–35]. To test for activity against blood-stage parasites in culture, available assays include antibody inhibition of erythrocyte invasion by merozoites [82], antibody opsonization enabling phagocytosis of merozoites [91], and antibody-dependent cellular inhibition (ADCI) of growth in the presence of monocytes [92]. Allele-specific inhibition has been demonstrated for antibodies to *P. falciparum* AMA1, an N-terminal part of MSP1, and *P. vivax* DBP [93–96], supporting the prediction that signatures of balancing selection on these merozoite antigens are driven by naturally acquired immune responses.

## Towards vaccine formulation

Design of malaria vaccines should incorporate discoveries from the above areas of research, although there remains no standardized means or structure for capturing these. Different alternative approaches will remain potentially viable. In particular, vaccines designed to prime immune responses in infants to specific targets of naturally acquired immunity need to either focus immune responses on conserved parts of the antigens or to elicit polyvalent responses that cover the natural antigenic diversity. If polymorphisms are clustered in regions of a primary sequence, it may be possible to use a relatively conserved part of the sequence, an approach that has been followed to focus on a C-terminal sequence of MSP3 of *P. falciparum* as a vaccine component [92]. When polymorphisms are broadly interspersed with conserved parts of sequences, for example within AMA1 or the *P. vivax* DBP, such dissection to remove polymorphic sites may not be possible, although particular formulations may still induce strong antibody responses to conserved epitopes [95,97]. Alternatively, multiple allele-specific antibodies may be generated to give protection against the range of allelic types circulating within populations, a ‘diversity-covering’ approach used for several vaccine formulations that have reached clinical trials, based on MSP1 [98], MSP2 [99], and AMA1 [100], as well as allelic combinations of both MSP1 and AMA1 together [101,102].

Antigenic diversity in relation to vaccination has been intensively studied for AMA1, in which the particular importance of a highly polymorphic amino acid position has been shown in several studies, including a clinical trial demonstrating allele-specific protection [103,104]. Experimental animal antibody inhibition of erythrocyte invasion in culture indicates that a multivalent vaccine containing between three and five different allelic types of AMA1 would cover most of the epitope polymorphism in this antigen [93,94]. For the polymorphic N-terminal region of MSP1, polyvalent hybrid proteins have been constructed that induce a wide range of antibody specificities which recognize the diverse natural allelic sequences [105,106]. In the cases of other antigens (the C-terminal region of MSP1 and the near-full length of MSP2 separately), for which known allelic sequences group into dimorphic allelic classes, two allelic types have been incorporated into formulations for clinical trial [98,99,101].

There remains no consensus template for an effective malaria vaccine, but the success of multivalent vaccines against viral and bacterial infections supports the rationale to characterize individual targets of immunity in detail to assist the design of subunit vaccines. Engineering of more effective immunogens is vital, and should involve modeled protein design to focus responses on important epitopes [107], as well as the use of the most-effective delivery systems [108]. Application of these principles, combined with lessons from empirical studies of a small number of malaria candidate antigens to date, should enable genome-scale studies on the parasite to be more systematically translated into the development of malaria vaccines.

## Figures and Tables

**Figure I:**
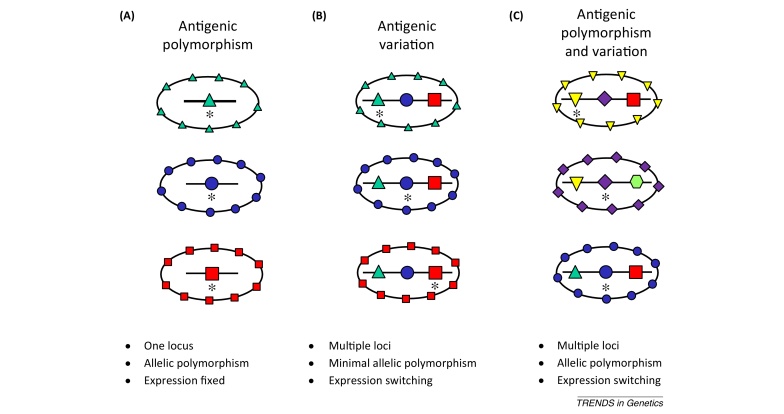
Parasites are depicted here as oval-shaped cells expressing a single antigen type on the surface, encoded by a gene at a particular chromosomal locus marked by a similar shape which is enlarged for visibility. Transcriptionally active loci are indicated with an asterisk. For absolute schematic simplicity, no cellular details (e.g., nucleus) are shown. **(A)** Allelic polymorphism of a single locus with fixed expression (as for many malaria vaccine candidates such as CSP, TRAP, MSP1, and AMA1). **(B)** Variation due to alternative expression of different loci with minimal allelic polymorphism (as for malaria parasite antigens including some of the EBA and Rh ligands using alternative erythrocyte receptors). **(C)** Variable expression of highly polymorphic loci contributing to a very extensive potential repertoire of diversity (as for parasite genes encoding PfEMP1, and for some of the CLAG/RhopH1 and MSP3-like proteins).

## Box 1 Malaria parasites – species and life-cycle targets for vaccination

The major malaria parasites of humans are *P. falciparum* and *P. vivax*. Vaccine development is generally considered separately for each of these because they are distantly related and have only limited antigenic cross-reactivity. Other human malaria parasites *P. malariae*, *P. ovale* (comprising biological species *P. o. curtisi* and *P. o. wallikeri*), and *P. knowlesi* (a zoonosis from macaque monkeys) are less common globally and are not considered for vaccine development. Malaria parasites are haploid throughout the cycle except for a brief diploid zygote stage after fertilization in the midgut of the mosquito. Phases of the parasite life cycle that are candidate targets for vaccination are:

*Pre-erythrocytic stage*. Invasive sporozoites inoculated by mosquitoes and subsequent development to large schizonts within the liver (taking ∼6 days in *P. falciparum*, and 10 days or longer for *P. vivax*) which do not cause disease but are a potential target to prevent blood stage infection.

*Asexual blood stage*. Causes all the symptoms of malaria as a result of significant parasitaemia achieved by repeated multiplication cycles (every ∼48 h for *P. falciparum* and *P. vivax*), with merozoite invasion followed by growth and replication of the parasite within the erythrocyte (forming early trophozoite ‘ring’ stages, late trophozoites, and schizonts) before the release of more merozoites.

*Sexual stage*. Male and female gametocytes in the blood are ingested by mosquitoes and transform into gametes and ensuing motile zygotes (ookinetes) which might be targeted by antibodies taken with the parasites into the blood meal.

## Box 2 Malaria parasite antigens

Abbreviated and full names of antigens with sequences incorporated in experimental vaccine trials already, and others mentioned in the article.

*In reported trials*

**Table:**

	
AMA1	Apical membrane antigen 1
CSP	Circumsporozoite protein
EBA175	Erythrocyte binding antigen 175 kDa
EXP-1	Exported protein-1
GLURP	Glutamate-rich protein
LSA-1	Liver stage antigen-1
LSA-3	Liver stage antigen-3
STARP	Sporozoite threonine-asparagine-rich protein
MSP1	Merozoite surface protein 1
MSP2	Merozoite surface protein 2
MSP3	Merozoite surface protein 3
Pfs25	*P. falciparum* sexual stage antigen 25 kDa
RESA	Ring-infected erythrocyte surface protein
SERA5	Serine-rich antigen 5
TRAP	Thrombospondin-related adhesive protein

*Others*

**Table:**

CLAG	Cytoadherence-linked asexual protein, also known as heavy molecular weight rhoptry protein family 1 (RhopH1)
DBP	Duffy-binding protein in *P. vivax* (homologous to *P. falciparum* EBA proteins)
EBA140	Erythrocyte binding antigen 140 kDa
EBL-1	Erythrocyte-binding-like protein 1
MSP6	Merozoite surface protein 6 (also known as MSP3.2, paralogous to MSP3 and MSPDBP)
MSPDBL1	Merozoite surface protein with Duffy-binding-like domain (also known as MSP3.4)
MSPDBL2	Merozoite surface protein with Duffy-binding-like domain (also known as MSP3.8)
MTRAP	Merozoite-specific TRAP homolog
PfEMP1	*P. falciparum* erythrocyte membrane protein 1 encoded by *var* genes
Rh4	Reticulocyte-binding-like protein homolog 4
Rh5	Reticulocyte-binding-like protein homolog 5
RIFIN	Repetitive interspersed family, gene product
Ripr	Rh5-interacting protein
S-Antigen	Heat-stable soluble antigen
STEVOR	Subtelomeric variant open reading frame, gene product
SURFIN	Surface-associated interspersed gene family product
VAR2CSA	A particular PfEMP1 that binds chondroitin sulfate A

## Box 3 Antigen gene polymorphism and expression variation

Some parasite antigens are encoded by a single gene with allelic polymorphism, and others are encoded by different paralogous genes that can switch transcriptional expression (Figure I).

## References

[1] Hoffman S.L. (1998). From genomics to vaccines: malaria as a model system. Nat. Med..

[2] White N.J. (2014). Malaria. Lancet.

[3] Moorthy V.S. (2012). Decision-making on malaria vaccine introduction: the role of cost-effectiveness analyses. Bull. World Health Organ..

[4] Hill A.V. (2011). Vaccines against malaria. Philos. Trans. R. Soc. Lond. B: Biol. Sci..

[5] Birkett A.J. (2013). Malaria vaccine R&D in the Decade of Vaccines: breakthroughs, challenges and opportunities. Vaccine.

[6] Gardner M.J. (2002). Genome sequence of the human malaria parasite *Plasmodium falciparum*. Nature.

[7] Carlton J.M. (2008). Comparative genomics of the neglected human malaria parasite *Plasmodium vivax*. Nature.

[8] Pain A. (2008). The genome of the simian and human malaria parasite *Plasmodium knowlesi*. Nature.

[9] Tachibana S. (2012). *Plasmodium cynomolgi* genome sequences provide insight into *Plasmodium vivax* and the monkey malaria clade. Nat. Genet..

[10] Otto T.D. (2014). The genomes of chimpanzee malaria parasites reveal possible pathways of adaptation to human hosts. Nat. Commun..

[11] Hall N. (2005). A comprehensive survey of the *Plasmodium* life cycle by genomic, transcriptomic, and proteomic analyses. Science.

[12] Kooij T.W. (2005). A *Plasmodium* whole-genome synteny map: indels and synteny breakpoints as foci for species-specific genes. PLoS Pathog..

[13] Frech C., Chen N. (2013). Variant surface antigens of malaria parasites: functional and evolutionary insights from comparative gene family classification and analysis. BMC Genomics.

[14] Jiang H. (2011). High recombination rates and hotspots in a *Plasmodium falciparum* genetic cross. Genome Biol..

[15] Nkhoma S.C. (2012). Close kinship within multiple-genotype malaria parasite infections. Proc. Biol. Sci..

[16] Manske M. (2012). Analysis of *Plasmodium falciparum* diversity in natural infections by deep sequencing. Nature.

[17] Bopp S.E. (2013). Mitotic evolution of *Plasmodium falciparum* shows a stable core genome but recombination in antigen families. PLoS Genet..

[18] Collins W.E., Jeffery G.M. (1999). A retrospective examination of sporozoite- and trophozoite-induced infections with *Plasmodium falciparum* in patients previously infected with heterologous species of *Plasmodium*: effect on development of parasitologic and clinical immunity. Am. J. Trop. Med. Hyg..

[19] Fowkes F.J. (2010). The relationship between anti-merozoite antibodies and incidence of *Plasmodium falciparum* malaria: a systematic review and meta-analysis. PLoS Med..

[20] Roestenberg M. (2011). Long-term protection against malaria after experimental sporozoite inoculation: an open-label follow-up study. Lancet.

[21] McCarthy J.S., Good M.F. (2010). Whole parasite blood stage malaria vaccines: a convergence of evidence. Hum. Vaccin..

[22] Seder R.A. (2013). Protection against malaria by intravenous immunization with a nonreplicating sporozoite vaccine. Science.

[23] Good M.F. (2013). Cross-species malaria immunity induced by chemically attenuated parasites. J. Clin. Invest..

[24] van Schaijk B.C. (2014). A genetically attenuated malaria vaccine candidate based on gene-deficient sporozoites. Elife.

[25] Mikolajczak S.A. (2014). A next-generation genetically attenuated *Plasmodium falciparum* parasite created by triple gene deletion. Mol. Ther..

[26] Yeh E., DeRisi J.L. (2011). Chemical rescue of malaria parasites lacking an apicoplast defines organelle function in blood-stage *Plasmodium falciparum*. PLoS Biol..

[27] Gisselberg J.E. (2013). The suf iron–sulfur cluster synthesis pathway is required for apicoplast maintenance in malaria parasites. PLoS Pathog..

[28] McAllister M.M. (2014). Successful vaccines for naturally occurring protozoal diseases of animals should guide human vaccine research. A review of protozoal vaccines and their designs. Parasitology.

[29] Schwartz L. (2012). A review of malaria vaccine clinical projects based on the WHO rainbow table. Malar. J..

[30] RTS,S Clinical Trials Partnership (2014). Efficacy and safety of the RTS,S/AS01 malaria vaccine during 18 months after vaccination: a phase 3 randomized, controlled trial in children and young infants at 11 African sites. PLoS Med..

[31] Hill A.V. (2010). Prime–boost vectored malaria vaccines: progress and prospects. Hum. Vaccin..

[32] Tamminga C. (2013). Human adenovirus 5-vectored *Plasmodium falciparum* NMRC-M3V-Ad-PfCA vaccine encoding CSP and AMA1 is safe, well-tolerated and immunogenic but does not protect against controlled human malaria infection. Hum. Vaccin. Immunother..

[33] Osier F.H. (2014). New antigens for a multicomponent blood-stage malaria vaccine. Sci. Transl. Med..

[34] Richards J.S. (2013). Identification and prioritization of merozoite antigens as targets of protective human immunity to *Plasmodium falciparum* malaria for vaccine and biomarker development. J. Immunol..

[35] Tetteh K.K. (2013). Analysis of antibodies to newly described *Plasmodium falciparum* merozoite antigens supports MSPDBL2 as a predicted target of naturally acquired immunity. Infect. Immun..

[36] Stanisic D.I. (2009). Immunoglobulin G subclass-specific responses against *Plasmodium falciparum* merozoite antigens are associated with control of parasitemia and protection from symptomatic illness. Infect. Immun..

[37] Tran T.M. (2013). An intensive longitudinal cohort study of Malian children and adults reveals no evidence of acquired immunity to *Plasmodium falciparum* infection. Clin. Infect. Dis..

[38] Trieu A. (2011). Sterile protective immunity to malaria is associated with a panel of novel *P. falciparum* antigens. Mol Cell Proteomics.

[39] Cheesman S. (2010). A single parasite gene determines strain-specific protective immunity against malaria: the role of the merozoite surface protein I. Int. J. Parasitol..

[40] Li J. (2011). Linkage maps from multiple genetic crosses and loci linked to growth-related virulent phenotype in *Plasmodium yoelii*. Proc. Natl. Acad. Sci. U.S.A..

[41] Hayton K. (2008). Erythrocyte binding protein PfRH5 polymorphisms determine species-specific pathways of *Plasmodium falciparum* invasion. Cell Host Microbe.

[42] Kinga Modrzynska K. (2012). Quantitative genome re-sequencing defines multiple mutations conferring chloroquine resistance in rodent malaria. BMC Genomics.

[43] Blake D.P. (2011). Genetic mapping identifies novel highly protective antigens for an apicomplexan parasite. PLoS Pathog..

[44] Roestenberg M. (2012). Comparison of clinical and parasitological data from controlled human malaria infection trials. PLoS ONE.

[45] Kaushansky A. (2014). Of men in mice: the success and promise of humanized mouse models for human malaria parasite infections. Cell. Microbiol..

[46] Pfander C. (2011). A scalable pipeline for highly effective genetic modification of a malaria parasite. Nat. Methods.

[47] Maier A.G. (2009). Malaria parasite proteins that remodel the host erythrocyte. Nat. Rev. Microbiol..

[48] Collins C.R. (2013). Robust inducible Cre recombinase activity in the human malaria parasite *Plasmodium falciparum* enables efficient gene deletion within a single asexual erythrocytic growth cycle. Mol. Microbiol..

[49] Ghorbal M. (2014). Genome editing in the human malaria parasite *Plasmodium falciparum* using the CRISPR–Cas9 system. Nat. Biotechnol..

[50] Baum J. (2009). Reticulocyte-binding protein homologue 5 – an essential adhesin involved in invasion of human erythrocytes by *Plasmodium falciparum*. Int. J. Parasitol..

[51] Crosnier C. (2011). Basigin is a receptor essential for erythrocyte invasion by *Plasmodium falciparum*. Nature.

[52] Bustamante L.Y. (2013). A full-length recombinant *Plasmodium falciparum* PfRH5 protein induces inhibitory antibodies that are effective across common PfRH5 genetic variants. Vaccine.

[53] Douglas A.D. (2014). Neutralization of *Plasmodium falciparum* merozoites by antibodies against PfRH5. J. Immunol..

[54] Williams A.R. (2012). Enhancing blockade of *Plasmodium falciparum* erythrocyte invasion: assessing combinations of antibodies against PfRH5 and other merozoite antigens. PLoS Pathog..

[55] Wright K.E. (2014). Structure of malaria invasion protein RH5 with erythrocyte basigin and blocking antibodies. Nature.

[56] Gomez-Escobar N. (2010). Erythrocyte invasion and merozoite ligand gene expression in severe and mild *Plasmodium falciparum* malaria. J. Infect. Dis..

[57] Hayton K. (2013). Various PfRH5 polymorphisms can support *Plasmodium falciparum* invasion into the erythrocytes of owl monkeys and rats. Mol. Biochem. Parasitol..

[58] Chen L. (2011). An EGF-like protein forms a complex with PfRh5 and is required for invasion of human erythrocytes by *Plasmodium falciparum*. PLoS Pathog..

[59] Bartholdson S.J. (2012). Semaphorin-7A is an erythrocyte receptor for P. falciparum merozoite-specific TRAP homolog, MTRAP. PLoS Pathog..

[60] Srinivasan P. (2014). Immunization with a functional protein complex required for erythrocyte invasion protects against lethal malaria. Proc. Natl. Acad. Sci. U.S.A..

[61] Gupta A.P. (2013). Dynamic epigenetic regulation of gene expression during the life cycle of malaria parasite *Plasmodium falciparum*. PLoS Pathog..

[62] Lopez-Barragan M.J. (2011). Directional gene expression and antisense transcripts in sexual and asexual stages of *Plasmodium falciparum*. BMC Genomics.

[63] Painter H.J. (2011). The Apicomplexan AP2 family: integral factors regulating *Plasmodium* development. Mol. Biochem. Parasitol..

[64] Guizetti J., Scherf A. (2013). Silence, activate, poise and switch! Mechanisms of antigenic variation in *Plasmodium falciparum*. Cell. Microbiol..

[65] Rovira-Graells N. (2012). Transcriptional variation in the malaria parasite *Plasmodium falciparum*. Genome Res..

[66] Amambua-Ngwa A. (2012). Population genomic scan for candidate signatures of balancing selection to guide antigen characterization in malaria parasites. PLoS Genet..

[67] Sander A.F. (2011). Positive selection of *Plasmodium falciparum* parasites with multiple var2csa-type PfEMP1 genes during the course of infection in pregnant women. J. Infect. Dis..

[68] Dahlback M. (2011). The chondroitin sulfate A-binding site of the VAR2CSA protein involves multiple N-terminal domains. J. Biol. Chem..

[69] Gangnard S. (2013). Structural and immunological correlations between the variable blocks of the VAR2CSA domain DBL6epsilon from two *Plasmodium falciparum* parasite lines. J. Mol. Biol..

[70] Nunes-Silva S. (2014). Llama immunization with full-length VAR2CSA generates cross-reactive and inhibitory single-domain antibodies against the DBL1X domain. Sci. Rep..

[71] Warimwe G.M. (2012). Prognostic indicators of life-threatening malaria are associated with distinct parasite variant antigen profiles. Sci. Transl. Med..

[72] Merrick C.J. (2012). Epigenetic dysregulation of virulence gene expression in severe *Plasmodium falciparum* malaria. J. Infect. Dis..

[73] Lavstsen T. (2012). *Plasmodium falciparum* erythrocyte membrane protein 1 domain cassettes 8 and 13 are associated with severe malaria in children. Proc. Natl. Acad. Sci. U.S.A..

[74] Claessens A. (2012). A subset of group A-like var genes encodes the malaria parasite ligands for binding to human brain endothelial cells. Proc. Natl. Acad. Sci. U.S.A..

[75] Turner L. (2013). Severe malaria is associated with parasite binding to endothelial protein C receptor. Nature.

[76] Smith J.D. (2013). Malaria's deadly grip: cytoadhesion of *Plasmodium falciparum* infected erythrocytes. Cell. Microbiol..

[77] Ghumra A. (2012). Induction of strain-transcending antibodies against Group A PfEMP1 surface antigens from virulent malaria parasites. PLoS Pathog..

[78] Niang M. (2014). STEVOR is a *Plasmodium falciparum* erythrocyte binding protein that mediates merozoite invasion and rosetting. Cell Host Microbe.

[79] Mwakalinga S.B. (2012). Expression of a type B RIFIN in *Plasmodium falciparum* merozoites and gametes. Malar. J..

[80] Tham W.H. (2012). Erythrocyte and reticulocyte binding-like proteins of *Plasmodium falciparum*. Trends Parasitol..

[81] Lopaticki S. (2011). Reticulocyte and erythrocyte binding-like proteins function cooperatively in invasion of human erythrocytes by malaria parasites. Infect. Immun..

[82] Healer J. (2013). Vaccination with conserved regions of erythrocyte-binding antigens induces neutralizing antibodies against multiple strains of *Plasmodium falciparum*. PLoS ONE.

[83] Weedall G.D., Conway D.J. (2010). Detecting signatures of balancing selection to identify targets of anti-parasite immunity. Trends Parasitol..

[84] Mobegi V.A. (2014). Genome-wide analysis of selection on the malaria parasite *Plasmodium falciparum* in West African populations of differing infection endemicity. Mol. Biol. Evol..

[85] Amambua-Ngwa A. (2012). SNP genotyping identifies new signatures of selection in a deep sample of West African *Plasmodium falciparum* malaria parasites. Mol. Biol. Evol..

[86] Park D.J. (2012). Sequence-based association and selection scans identify drug resistance loci in the *Plasmodium falciparum* malaria parasite. Proc. Natl. Acad. Sci. U.S.A..

[87] Miotto O. (2013). Multiple populations of artemisinin-resistant *Plasmodium falciparum* in Cambodia. Nat. Genet..

[88] Neafsey D.E. (2012). The malaria parasite *Plasmodium vivax* exhibits greater genetic diversity than *Plasmodium falciparum*. Nat. Genet..

[89] Chenet S.M. (2012). Genetic diversity and population structure of genes encoding vaccine candidate antigens of *Plasmodium vivax*. Malar. J..

[90] Iqbal Z. (2012). De novo assembly and genotyping of variants using colored de Bruijn graphs. Nat. Genet..

[91] Osier F.H. (2014). Opsonic phagocytosis of *Plasmodium falciparum* merozoites: mechanism in human immunity and a correlate of protection against malaria. BMC Med..

[92] Jepsen M.P. (2013). The malaria vaccine candidate GMZ2 elicits functional antibodies in individuals from malaria endemic and non-endemic areas. J. Infect. Dis..

[93] Miura K. (2013). Overcoming allelic specificity by immunization with five allelic forms of *Plasmodium falciparum* apical membrane antigen 1. Infect. Immun..

[94] Drew D.R. (2012). Defining the antigenic diversity of *Plasmodium falciparum* apical membrane antigen 1 and the requirements for a multi-allele vaccine against malaria. PLoS ONE.

[95] Ntumngia F.B. (2012). Conserved and variant epitopes of *Plasmodium vivax* Duffy binding protein as targets of inhibitory monoclonal antibodies. Infect. Immun..

[96] Galamo C.D. (2009). Anti-MSP1 block 2 antibodies are effective at parasite killing in an allele-specific manner by monocyte-mediated antibody-dependent cellular inhibition. J. Infect. Dis..

[97] Dutta S. (2013). Overcoming antigenic diversity by enhancing the immunogenicity of conserved epitopes on the malaria vaccine candidate apical membrane antigen-1. PLoS Pathog..

[98] Ellis R.D. (2010). Phase 1 trial of the *Plasmodium falciparum* blood stage vaccine MSP1(42)-C1/Alhydrogel with and without CPG 7909 in malaria naive adults. PLoS ONE.

[99] McCarthy J.S. (2011). A phase 1 trial of MSP2-C1, a blood-stage malaria vaccine containing 2 isoforms of MSP2 formulated with Montanide(R) ISA 720. PLoS ONE.

[100] Sagara I. (2009). A randomized controlled phase 2 trial of the blood stage AMA1-C1/Alhydrogel malaria vaccine in children in Mali. Vaccine.

[101] Elias S.C. (2013). Assessment of immune interference, antagonism, and diversion following human immunization with biallelic blood-stage malaria viral-vectored vaccines and controlled malaria infection. J. Immunol..

[102] Ellis R.D. (2012). Phase 1 study in malaria naive adults of BSAM2/Alhydrogel(R)+CPG 7909, a blood stage vaccine against *P. falciparum* malaria. PLoS ONE.

[103] Thera M.A. (2011). A field trial to assess a blood-stage malaria vaccine. N. Engl. J. Med..

[104] Ouattara A. (2013). Molecular basis of allele-specific efficacy of a blood-stage malaria vaccine: vaccine development implications. J. Infect. Dis..

[105] Cowan G.J. (2011). A malaria vaccine based on the polymorphic block 2 region of MSP-1 that elicits a broad serotype-spanning immune response. PLoS ONE.

[106] Tetteh K.K., Conway D.J. (2011). A polyvalent hybrid protein elicits antibodies against the diverse allelic types of block 2 in *Plasmodium falciparum* merozoite surface protein 1. Vaccine.

[107] Correia B.E. (2014). Proof of principle for epitope-focused vaccine design. Nature.

[108] Koff W.C. (2013). Accelerating next-generation vaccine development for global disease prevention. Science.

